# Risk factor screening and prediction modeling of gastrointestinal adverse reactions caused by GLP-1RAs

**DOI:** 10.3389/fendo.2024.1502050

**Published:** 2024-12-05

**Authors:** Rui Gao, Yue Li, Anan Li, Penglin Zhou, Huiying Zong, Yan Li

**Affiliations:** ^1^ Department of Clinical Pharmacy, The First Affiliated Hospital of Shandong First Medical University, Jinan, China; ^2^ Department of Medicine, Qilu Institute of Technology, Jinan, China; ^3^ Department of Pharmacy, Shandong University of Traditional Chinese Medicine, Jinan, China

**Keywords:** type 2 diabetes mellitus, glucagon-like peptide-1 receptor agonists, gastrointestinal side effects, risk factors, nomogram

## Abstract

**Objective:**

This study aimed to explore the risk factors for gastrointestinal side effects (GISEs) in patients with type 2 diabetes mellitus (T2DM) during treatment with glucagon-like peptide-1 receptor agonists (GLP-1RAs) based on real-world data and to develop a prediction model for GLP-1RA-related GISEs.

**Methods:**

A total of 855 patients who attended the First Affiliated Hospital of Shandong First Medical University from January 2020 to May 2023 were selected as the study participants, who were divided into the training set (598 cases) and the validation set (297 cases) using a simple random sampling method at a ratio of 7:3. The general information and biochemical indicators of the participants were collected to assess the risk factors for GLP-1RA-related GISEs, and multifactorial logistic regression analysis was used to obtain the best predictors. A nomogram prediction model was constructed. The Hosmer–Lemeshow test was used to assess the differentiation and calibration of the nomogram model, and decision curve analysis (DCA) was used to evaluate the clinical utility of the model.

**Results:**

Age, gender, history of gastrointestinal disorders, and number of combined oral medications were found as risk factors for the occurrence of GISEs in patients with T2DM using GLP-1RAs (*p* < 0.05). The nomogram prediction model based on these four factors had good discriminability (AUC values of the training and validation sets of 0.855 and 0.836, respectively) and accuracy (Hosmer–Lemeshow test: *p* > 0.05 for the validation set). DCA showed that the prediction model curve had clinical utility in the threshold probability interval of >5%.

**Conclusions:**

The established nomogram model has an excellent predictive effect on GISEs induced by GLP-1RAs in patients with T2DM.

## 1 Introduction

The prevalence of type 2 diabetes mellitus (T2DM) has been on the rise globally in recent years, with data from the International Diabetes Federation (IDF) showing that there will be 783.2 million people (12.2%) living with diabetes by 2045 (1), of which T2DM will account for approximately 90% (2). Glucagon-like peptide-1 receptor agonists (GLP-1RAs) were approved for T2DM treatment in 2005. Now, the American Diabetes Association (ADA) has recommended in the *Standards of Care in Diabetes* the addition of GLP-1RAs and/or SGLT2 inhibitors (SGLT2i) to the treatment plan for patients with T2DM who have been diagnosed with or are at high risk of atherosclerotic cardiovascular disease (ASCVD) (3). Cardiovascular outcome studies have demonstrated that GLP-1RAs are effective in preventing cardiovascular events (such as acute myocardial infarction or stroke), reducing associated mortality (4–7), and in helping to prevent the progression of renal complications in patients with T2DM (5–8). GLP-1RAs are recommended as the preferred injectable hypoglycemic therapy for T2DM, even superior to insulin therapy (9), due to their definite cardiac and renal benefits.

However, the occurrence of gastrointestinal side effects (GISEs) has challenged the application of GLP-1RAs, which were clinically characterized by symptoms such as nausea (42.23%), vomiting (21.90%), diarrhea (21.93%), and constipation (8.41%) (10), and the incidence of severe adverse reactions was 15.43% (10). It has been shown that GISEs are associated with central and peripheral glucagon-like peptide-1 receptor (GLP-1R) activation (11). These adverse effects caused by treatment with GLP-1RAs are generally short-lived and gradually subside after a few weeks of treatment and therefore can be tolerated by most patients. Nevertheless, there are a few patients who are unable to tolerate severe GISEs caused by GLP-1RAs, resulting in the discontinuation of GLP-1RA treatment, leading to delays in the disease control and to financial losses. The study results obtained by Zhang et al. (12) showed that GLP-1RAs had the highest risk of intolerable gastrointestinal adverse events among hypoglycemic drugs, with a cumulative percentage area under the ranking curve of 91.8. The severity of GISEs varies among GLP-1RAs (10). Some studies have confirmed that the risk factors for GISEs caused by liraglutide may include the co-application of α-glucosidase inhibitors or metformin, history of gastrointestinal disorders, gender, age, and elevated levels of thyroid-stimulating hormone (TSH) (13–15). However, until now, there has been a lack of well-designed studies to validate the risk factors for GISEs caused by GLP-1RAs; thus, there is an urgent need to establish a risk prediction model for the identification of the high-risk population. Therefore, the main purposes of this study were to investigate the risk factors for GLP-1RA-related GISEs and to develop an effective prediction model so as to provide a basis for the individualization of GLP-1RAs.

## 2 Methods

### 2.1 Research design

#### 2.1.1 Study participants and data sources

The data used in the study were derived from the healthcare big data platform and the electronic medical record system of the First Affiliated Hospital of Shandong First Medical University (Shandong Provincial Qianfoshan Hospital). Patients with T2DM who received GLP-1RAs for the first time between January 1, 2020, and May 31, 2023, were included. T2DM was diagnosed according to the diagnostic criteria in the Chinese Guidelines for the Prevention and Control of Type 2 Diabetes Mellitus (2020), as shown in **Supplementary Table S1**. Patient information including the demographic characteristics, co-administered medications, laboratory data, history of gastrointestinal disorders, and clinical diagnosis were collected for further analysis.

#### 2.1.2 Inclusion and exclusion criteria

The inclusion criteria for study participation were: 1) patients with T2DM; 2) aged 18 years and older; 3) patients with poor glycemic control and first-time treatment with GLP-1RAs; and 4) patients with well-established demographics, laboratory data, and medical and medication history. The exclusion criteria were: 1) patients with malignant tumors; 2) patients with a dialysis or renal transplantation history; 3) patients with end-stage diseases; 4) patients with a history of drug abuse or with severe pancreatic diseases; 5) patients with severe gastrointestinal dysfunction; 6) patients with severe hepatic or renal insufficiency; and 7) women who are pregnant or breastfeeding.

#### 2.1.3 Determination of the correlation of adverse drug reactions and GLP-1RAs

The symptoms of GISEs include nausea, vomiting, abdominal pain, diarrhea, reflux, flatulence, and other similar conditions. The correlation between the use of GLP-1RAs and the occurrence of GISEs was evaluated according to Naranjo’s scale (16) (a Naranjo score ≥5 was considered as a GLP-1RA-related GISE). The Naranjo’s scoring criteria are shown in **Supplementary Table S2**.

### 2.2 Data processing

A simple random sampling method was used to divide the study population into a training set (598 cases) and a validation set (297 cases) at a ratio of 7:3 for analysis. All statistical analyses were performed in R software (version 3.6.3). The graphics were drawn using GraphPad Prism 9.0.0 software. Categorical variables were reported as frequencies and percentages, normally distributed continuous variables were described as the mean ± standard deviation, and non-normally distributed continuous variables were described as median (interquartile range). For the data statistics, a *t*-test was used for normally distributed variables, the Wilcoxon test for non-normally distributed variables, and the chi-square test for categorical variables (i.e., sex, history of gastrointestinal disorders, and smoking history). Binary logistic regression analysis was used to screen the predictors and to construct a nomogram prediction model. Moreover, receiver operating characteristic (ROC) curves were plotted, and the Hosmer–Lemeshow test was used to assess the discriminability and calibration of the prediction model. Finally, decision curve analysis (DCA) was used in order to assess the clinical utility of the model.

## 3 Results

### 3.1 Demographic characteristics of the study participants

**Figure 1:**
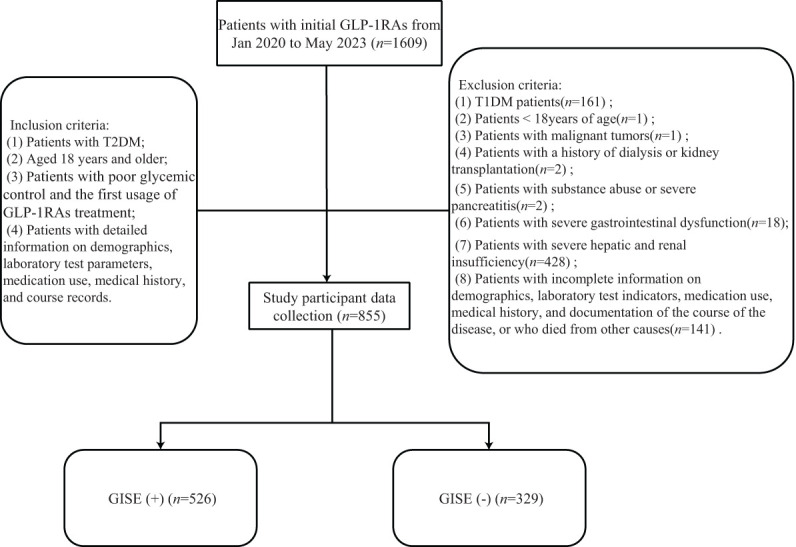
Participants inclusion flowchart. *GLP-1RAs*, glucagon-like peptide-1 receptor agonists; *GISE*, gastrointestinal side effect.

A total of 855 patients with T2DM were included in the present study, of whom 636 (74.39%) received injections of liraglutide, 137 (16.02%) of polyethylene glycol loxenatide, 44 (5.15%) of semaglutide, 23 (2.69%) of exenatide, 3 (0.35%) of dulaglutide, and 12 (1.40%) of lixisenatide. The research group included 624 (72.98%) men and 231 (27.02%) women, aged 54 years (44–62 years), with duration of diabetes mellitus of 4.00 years (0.15–10.00 years). The glycosylated hemoglobin (HbA_1c_) level was 8.40% (7.30%–9.90%), while the fasting blood glucose was 7.46 ±.75 mmol/L. A total of 526 (61.52%) of all enrolled patients developed GISEs, of which 14 (1.64%) discontinued GLP-1RA treatment due to intolerance to GISEs. The participant inclusion process is shown in **Figure 1**.

### 3.2 Univariate analysis of the clinical variables

Only the history of gastrointestinal disorders and diastolic blood pressure (DBP) were statistically different between the two groups in the training and validation sets (*p* < 0.05) (**Table 1**). The results of unifactorial screening based on the training set data are shown in **Table 2**. Significant differences were found between patients developing GISEs and those without GISEs in terms of age, gender, duration of diabetes, history of gastrointestinal disorders, smoking history, number of combined oral drugs (the types of combined oral medications are shown in **Supplementary Table S3**), body mass index (BMI), DBP, free triiodothyronine (FT_3_), free thyroxine (FT_4_), alanine aminotransferase (ALT), total bilirubin (TBIL), uric acid (UA), and estimated glomerular filtration rate (eGFR) (*p* < 0.05). However, family history of gastrointestinal disorders, history of alcoholism, heart rate (HR), systolic blood pressure (SBP), fasting blood glucose (FBG), glycated hemoglobin (HbA_1c_), total cholesterol (TC), triglycerides (TG), high-density lipoprotein cholesterol (HDL-C), low-density lipoprotein cholesterol (LDL-C), thyroid-stimulating hormone (TSH), fasting insulin (FINS), aspartate aminotransferase (AST), lipid profile, creatinine (enzyme assay) (Cr), β_2_-microglobulin (β_2_-M), red blood cell count (RBC), white blood cell count (WBC), and platelet count (PLT) were not significantly correlated with GISEs (*p* > 0.05).

**Table 1:** Results of the baseline feature comparison between the training and validation sets.

	Training set (*n* = 598)	Validation set (*n* = 257)	*p*-value
Age (years)	53 (45–63)	53 (44–60)	0.104
Gender (% women)	168 (28.09)	63 (24.51)	0.280
Duration of diabetes (years)	4.00 (0.15–10.00)	5.00 (0.42–10.00)	0.811
Family history of gastrointestinal disorders, *n* (%)	30 (5.02)	12 (4.67)	0.829
History of gastrointestinal disorders, *n* (%)	136 (22.74)	41 (15.95)	0.025^*^
Smoking history, *n* (%)	230 (38.46)	113 (43.97)	0.190
History of alcoholism, *n* (%)	191 (31.94)	92 (35.80)	0.531
No. of combined oral drugs	2 (2–4)	2 (2–4)	0.328
SBP (mmHg)	132.41 ± 14.55	134.10 ± 13.91	0.118
DBP (mmHg)	79.96 (73.41–86.17)	81.16 (75.97–87.19)	0.034^*^
HR (bpm)	82.87 ± 16.96	83.50 ± 13.44	0.961
BMI (kg/m^2^)	28.04 (25.93–30.48)	28.15 (26.05–30.69)	0.693
FBG (mmol/L)	7.47 ± 1.86	7.44 ± 1.49	0.894
FINS (μIU/ml)	8.70 (4.62–15.21)	9.77 (5.47–16.16)	0.350
HbA_1c_ (%)	8.40 (7.30–9.80)	8.45 (7.38–10.10)	0.398
TC (mmol/L)	13.86 (2.10–20.17)	14.68 (2.27–19.86)	0.560
TG (mmol/L)	2.86 (0.24–4.56)	0.43 (0.23–4.65)	0.945
HDL-C (mmol/L)	1.03 (0.91–1.17)	1.04 (0.90–1.17)	0.969
LDL-C (mmol/L)	2.58 ± 0.86	2.58 ± 0.87	0.966
TSH (μIU/ml)	1.83 (1.29–2.63)	1.63 (1.24–2.37)	0.060
FT_3_ (pmol/L)	4.65 (4.11–5.08)	4.62 (4.24–5.10)	0.361
FT_4_ (pmol/L)	18.52 (16.88–20.25)	18.38 (16.66–20.20)	0.668
ALT (U/L)	25.10 (18.50–38.70)	26.10 (18.30–40.15)	0.927
AST (U/L)	18.70 (14.70–24.82)	19.30 (14.70–25.20)	0.660
TBIL (μmol/L)	10.70 (8.10–14.62)	11.30 (8.30–14.20)	0.619
Cr (μmol/L)	67.00 (58.00–77.00)	66.00 (57.00–74.50)	0.214
Urea/Cr (ratio)	0.08 (0.07–0.10)	0.08 (0.07–0.10)	0.866
β_2_-M (mg/L)	1.63 (1.47–1.91)	1.63 (1.44–1.91)	0.259
eGFR (ml/min/1.73 m^2^)	107.59 (98.07–117.80)	109.14 (102.03–118.16)	0.163
UA (μmol/L)	340.00 (278.00–392.00)	331.50 (283.00–397.50)	0.914
RBC (×10^12^/L)	4.14 (2.00–5.13)	4.19 (2.00–5.00)	0.633
WBC (×10^9^/L)	6.34 (5.40–7.56)	6.37 (5.32–7.68)	0.993
PLT (×10^9^/L)	221.00 (184.00–262.00)	214.00 (183.00–252.00)	0.375

Variables are expressed as the mean ± standard deviation or percentage.

*SBP*, systolic blood pressure; *DBP*, diastolic blood pressure; *HR*, heart rate; *BMI*, body mass index; *FBG*, fasting blood glucose; *FINS*, fasting insulin; *HbA_1c_*, glycated hemoglobin; *TC*, total cholesterol; *TG*, triglycerides; *HDL-C*, high-density lipoprotein cholesterol; *LDL-C*, low-density-lipoprotein cholesterol; *TSH*, thyroid-stimulating hormone; *FT_3_*, free triiodothyronine; *FT_4_*, free thyroxine; *ALT*, alanine aminotransferase; *AST*, aspartate aminotransferase; *eGFR*, estimated glomerular filtration rate; *Cr*, creatinine; *Urea/Cr*, urea-to-creatinine ratio; *β_2_-M*, β_2_-microglobulin; *TBIL*, total bilirubin; *UA*, uric acid; *RBC*, red blood cell count; *WBC*, white blood cell count; *PLT*, platelet count.

*p < 0.05.

**Table 2:** Demographic information and metabolic and biochemical indices of patients in the training set with and without gastrointestinal adverse effects during the use of glucagon-like peptide-1 receptor agonists.

	GISE(−) (*n* = 220)	GISE(+) (*n* = 378)	*p*-value
Age (years)	48 (39–57)	57 (51–65)	<0.001*
Gender (% women)	40 (18.18)	128 (33.86)	<0.001*
Duration of diabetes (years)	1.00 (0.00–8.00)	6.00 (0.50–12.00)	<0.001*
Family history of gastrointestinal disorders, *n* (%)	7 (3.18)	23 (6.08)	0.117
History of gastrointestinal disorders, *n* (%)	21 (9.55)	115 (30.42)	<0.001*
Smoking history, *n* (%)	98 (44.55)	132 (34.92)	0.013*
History of alcoholism, *n* (%)	82 (37.27)	109 (28.84)	0.065
No. of combined oral drugs	1 (0–3)	3 (2–5)	<0.001*
BMI (kg/m^2^)	28.68 (26.14–31.09)	27.72 (25.66–30.17)	0.005*
HR (bpm)	83.44 ± 28.35	82.67 ± 13.94	0.950
SBP (mmHg)	132.26 ± 14.69	132.50 ± 14.49	0.848
DBP (mmHg)	81.47 (74.31–87.70)	79.00 (73.30–85.00)	0.013*
FBG (mmol/L)	7.41 ± 1.85	7.52 ± 1.87	0.710
HbA_1c_ (%)	8.40 (7.30–9.90)	8.40 (7.32–9.70)	0.811
TG (mmol/L)	3.28 (0.24–4.65)	2.67 (0.24–4.46)	0.625
HDL-C (mmol/L)	1.04 (0.91–1.16)	1.03 (0.92–1.17)	0.812
LDL-C (mmol/L)	2.60 ± 0.78	2.57 ± 0.91	0.745
TC (mmol/L)	13.34 (2.10–19.80)	14.09 (2.09–20.28)	0.748
TSH (μIU/mL)	1.74 (1.25–2.62)	1.90 (1.30–2.63)	0.451
FT_3_ (pmol/L)	4.76 (4.23–5.28)	4.56 (4.02–4.96)	<0.001*
FT_4_ (pmol/L)	18.95 (17.18–20.43)	18.24 (16.74–20.10)	0.046^*^
FINS (μIU/mL)	9.20 (4.15–15.46)	8.52 (5.00–15.15)	0.824
ALT (U/L)	26.40 (20.22–44.48)	24.50 (17.60–4.40)	0.028*
AST (U/L)	19.50 (15.75–27.10)	18.00 (14.25–23.70)	0.053
eGFR (ml/min/1.73 m^2^)	110.98 (100.38–120.94)	104.93 (96.97–114.70)	<0.001*
Cr (μmol/L)	69.00 (58.00–79.00)	67.00 (57.00–75.00)	0.224
Urea/Cr (ratio)	0.08 (0.06–0.10)	0.08 (0.07–0.09)	0.190
β_2_-M (mg/L)	1.60 (1.42–1.88)	1.68 (1.49–1.96)	0.068
TBIL (μmol/L)	12.00 (8.65–15.40)	10.30 (8.03–13.80)	0.024*
UA (μmol/L)	360.50 (291.00–408.00)	320.00 (271.00–383.50)	0.013*
RBC (×10^12^/L)	4.35 (2.00–5.11)	4.00 (2.00–5.14)	0.461
WBC (×10^9^/L)	6.25 (5.41–7.69)	6.42 (5.39–7.43)	0.829
PLT (×10^9^/L)	221.00 (190.00–255.50)	220.50 (183.25–263.00)	0.733

Variables are expressed as the mean ± standard deviation or percentage.

*SBP*, systolic blood pressure; *DBP*, diastolic blood pressure; *HR*, heart rate; *BMI*, body mass index; *FBG*, fasting blood glucose; *FINS*, fasting insulin; *HbA_1c_*, glycated hemoglobin; *TC*, total cholesterol; *TG*, triglycerides; *HDL-C*, high-density lipoprotein cholesterol; *LDL-C*, low-density lipoprotein cholesterol; *TSH*, thyroid-stimulating hormone; *FT_3_*, free triiodothyronine; *FT_4_*, free thyroxine; *ALT*, alanine aminotransferase; *AST*, aspartate aminotransferase; *eGFR*, estimated glomerular filtration rate; *Cr*, creatinine; *Urea/Cr*, urea-to-creatinine ratio; *β_2_-M*, β_2_-microglobulin; *TBIL*, total bilirubin; *UA*, uric acid; *RBC*, red blood cell count; *WBC*, white blood cell count; *PLT*, platelet count.

*p < 0.05.

### 3.3 Multifactor logistic regression analysis of the significant variables

The stepwise method of binary logistic regression was used to assess whether the significant variables in the univariate analysis were associated with GISEs (**Table 3**). Preliminary analysis of logistic regression showed that age [adjusted OR = 1.06 (1.02–1.09)], gender [adjusted OR = 2.86 (1.47–5.60)], number of combined oral drugs [adjusted OR = 1.94 (1.63–2.31)], history of gastrointestinal disorders [adjusted OR = 4.92 (2.39–10.12)], eGFR [adjusted OR = 1.02 (1.00–1.04)], and BMI [adjusted OR = 1.06 (0.99–1.14)]. The *p*-values for eGFR and BMI were greater than 0.05 and not statistically significant. In the final regression model, age [adjusted OR = 1.05 (1.04–1.07)], gender [adjusted OR = 2.32 (1.42–3.80)], number of combined oral drugs [adjusted OR = 1.84 (1.62–2.10)], and history of gastrointestinal disorders [adjusted OR = 6.22 (3.49–11.11)] were associated with GISEs caused by GLP-1RAs.

**Table 3:** Multifactorial logistic regression analysis of the gastrointestinal adverse reactions caused by glucagon-like peptide-1 receptor agonists.

	Primary model			Final model		
	*β*	OR (95%CI)	*p*-value	*β*	OR (95%CI)	*p*-value
Gender (% women)	1.05	2.86 (1.47–5.60)	0.002	0.84	2.32 (1.42–3.80)	<0.001
History of gastrointestinal disorders	1.59	4.92 (2.39–10.12)	<0.001	1.83	6.22 (3.49–11.11)	<0.001
Age (years)	0.05	1.06 (1.02–1.09)	<0.001	0.05	1.05 (1.04–1.07)	<0.001
No. of combined oral drugs	0.66	1.94 (1.63–2.31)	<0.001	0.61	1.84 (1.62–2.10)	<0.001
eGFR (ml/min/1.73 m^2^)	0.02	1.02 (1.00–1.04)	0.128			
BMI (kg/m^2^)	0.06	1.06 (0.99–1.14)	0.089			

### 3.4 Model development

**Figure 2:**
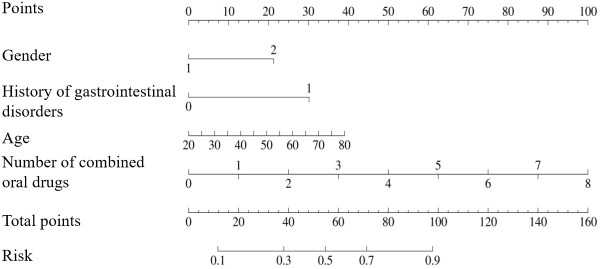
Nomogram including age, gender, number of combined oral drugs, and history of gastrointestinal disorders for the risk of gastrointestinal side effects in patients using glucagon-like peptide-1 receptor agonists.

The GLP-1RA-related GISEs in patients with T2DM were predicted based on four factors (i.e., age, gender, number of combined oral medications, and history of gastrointestinal disorders), which were plotted as a nomogram (**Figure 2**). According to the nomogram model, the effect of each variable on GISEs was reflected in the respective row lengths and corresponding scores, and the total score of the model was obtained by adding the scores of each factor. The probability that the reference total score corresponds to the risk of GISEs was the risk of GISEs for a specific patient.

### 3.5 Evaluation of the predictive efficiency and clinical applicability of the model

**Figure 3:**
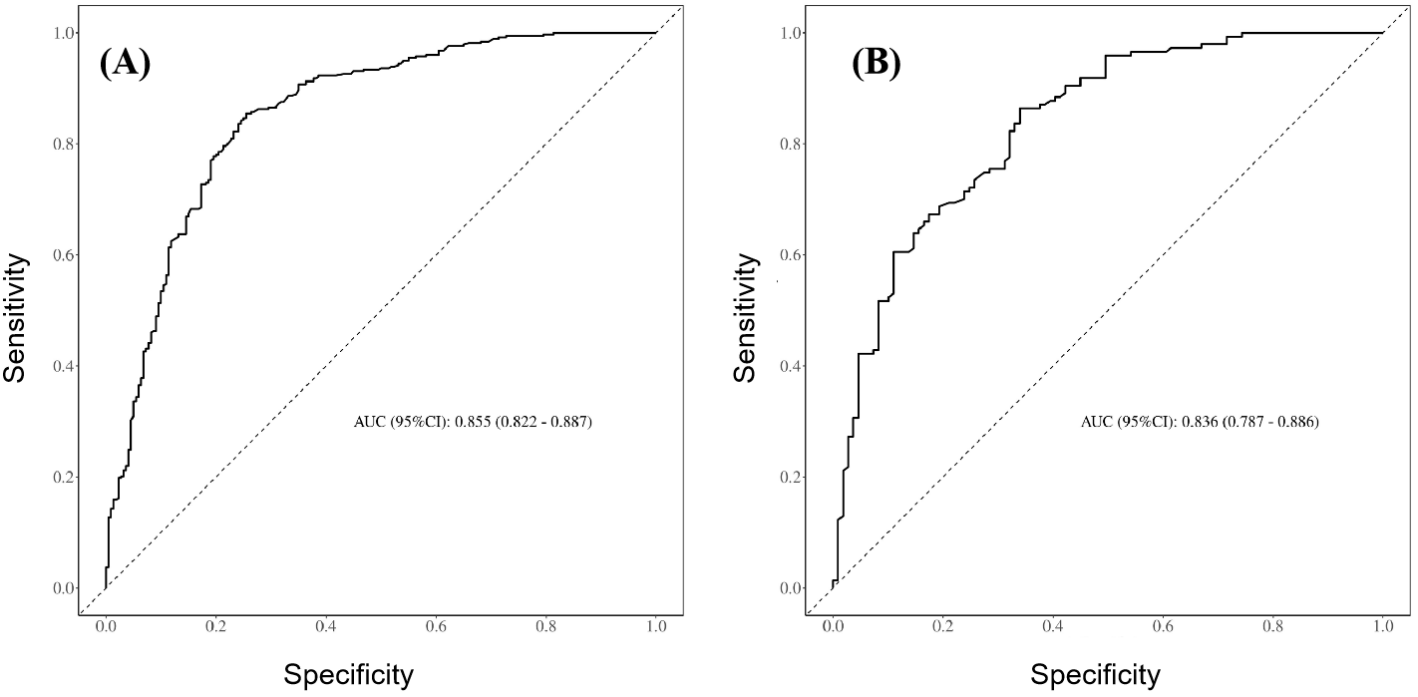
**(A)** Receiver operating characteristic (ROC) curves for the training set of the prediction model. **(B)** ROC curves for the validation set of the prediction model.

**Figure 4:**
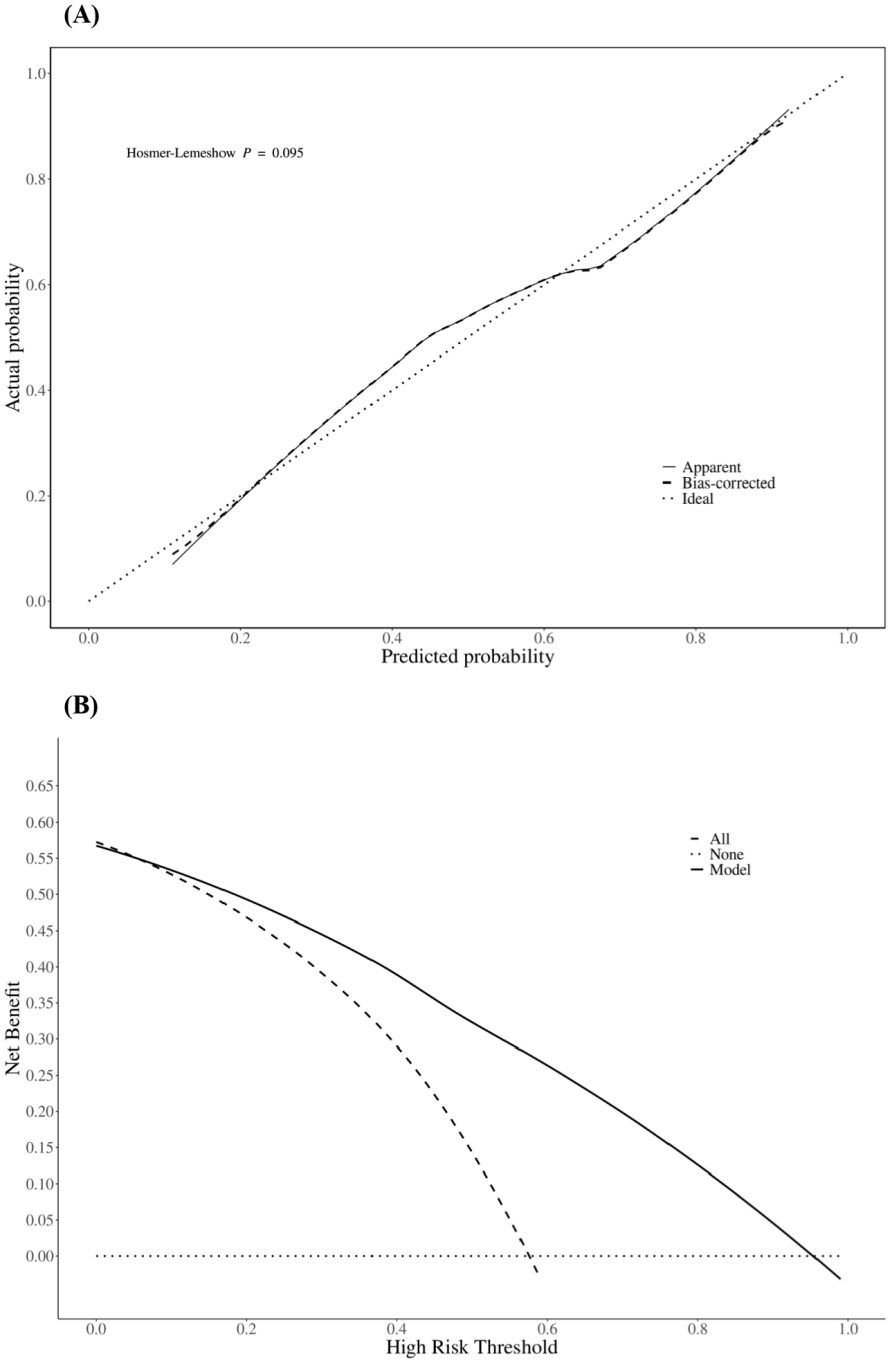
Calibration curves **(A)** and decision curves **(B)** of the nomogram predicting the risk of gastrointestinal side effect (GISE) occurrence.

The prediction performance of the nomogram was evaluated using ROC curves (the AUC values for the training and validation sets were 0.855 and 0.836, respectively), as shown in **Figures 3A, B**, respectively, indicating that the column-line diagram prediction model had good prediction ability. The calibration curves revealed that the predicted probabilities were generally consistent with the observed probabilities, indicating that the model had good goodness-of-fit (Hosmer–Lemeshow test: *p* = 0.095) (**Figure 4A**). The DCA curves demonstrated that screening with the GISE risk nomogram model at thresholds greater than 5% resulted in a high net clinical benefit (**Figure 4B**).

## 4 Discussion

The main finding of the present study is that age, gender, number of combined oral medications, and history of gastrointestinal disorders are risk factors for GISEs caused by GLP-1RAs. A nomogram model was used to establish a risk prediction model for the clinical application of GLP-1RAs, which is of great significance for the safety and individualized administration of GLP-1RAs in patients with T2DM.

A previous study found that aging is associated with an increased incidence of liraglutide-related GISEs (14), which is consistent with the outcomes of this study. The reasons may be that, with increased age, the gastrointestinal tract undergoes the following physiological degenerative changes: a) decreased resistance of the gastric mucosa to damages, which leads to an increased risk of gastrointestinal ulcer disease; b) gradually decreased secretion of gastric acid and pepsin, resulting in a decline in digestive function and, thus, an increased risk of functional digestive malfunction; and c) reduced water absorption capacity of the large intestine and rectum and a weakened motility, thus making it susceptible to swelling and constipation (17). Therefore, the use of GLP-1RAs should be taken with caution in elderly patients.

The number of combined oral medications is also a risk factor for the occurrence of GISEs, probably due to the fact that the greater the number of combined medications, the more complex the drug interactions *in vivo* (18). In addition, more oral medications have a superimposed gastrointestinal irritant effect that increases the risk of developing GISEs. Some studies have reported that 6%–30% of adverse drug reactions are caused by drug–drug interactions (19), particularly 5%–9% of adverse drug reactions in hospitalized patients (19), and that inappropriate combinations of medications could also lead to the enhanced side effects of specific medications (18), which could increase the risk of gastrointestinal adverse reactions in patients with T2DM treated with GLP-1RAs. A history of gastrointestinal disorders could also contribute to the development of GISEs, although gastrointestinal disorders are not a contraindication to the use of GLP-1RAs and comprised the risk factors for the development of GISEs in our study (*p* < 0.001). This might be due to the fact that GLP-1RAs have a delaying effect on gastrointestinal emptying, which could exacerbate gastrointestinal disorders or predispose to the development of prior gastrointestinal disorders. Therefore, patients with a history of gastrointestinal disorders are advised to use GLP-1RAs with caution in order to avoid poor tolerance and compliance.

In this study, the first nomogram prediction model was constructed to predict the risk of GLP-1RA-associated GISEs in patients with T2DM. The AUC values of both the training and test sets indicated that the model had high validity and accuracy in predicting GISEs, which had certain practical application value. The calibration curve showed that the predicted probability of the model was in good conformity with the actual probability. However, this study still has some limitations. As a cross-sectional study, we used data from patients during their hospitalization to analyze the status for a short period after the use of the target medication, and there was no information on the long-term follow-up of outpatients and inpatients, which could have some confounding factors. In addition, documentation of the GISEs was taken from the medical records, which might have some factual bias.

In summary, this study explores and establishes a convenient and practical nomogram model for predicting the risk of GISEs after the use of GLP-1RAs in patients with T2DM, which provides early warning information for patients to understand the risk of developing GISEs as early as possible and to take timely and appropriate interventions.

## Data Availability

The raw data supporting the conclusions of this article will be made available by the authors, without undue reservation.
