# Supplementary material for: Risk factor screening and prediction modeling of gastrointestinal adverse reactions caused by GLP-1RAs
Source: Front Endocrinol (Lausanne). 2024 Dec 5;15:1502050. doi: 10.3389/fendo.2024.1502050 (PMC11664219; doi:10.3389/fendo.2024.1502050)
Supplement: Supplementary file 1 [file Table1.doc]

Classification of glucose metabolic status

| glucose metabolic state | Intravenous plasma glucose | |
| --- | --- | --- |
|  | fasting blood glucose | Glucose 2h after sugar load |
| normoglycemia | ＜6.1 | ＜7.8 |
| Impaired fasting glucose | ≥6.1，＜7.0 | ＜7.8 |
| impaired glucose tolerance | ＜7.0 | ≥7.8，＜11.1 |
| diabetes | ≥7.0 | ≥11.1 |

Type 2 diabetes: Varying degrees of β-cell dysfunction and insulin resistance, often associated with overweight and obesity.
