# Supplementary material for: Risk factor screening and prediction modeling of gastrointestinal adverse reactions caused by GLP-1RAs
Source: Front Endocrinol (Lausanne). 2024 Dec 5;15:1502050. doi: 10.3389/fendo.2024.1502050 (PMC11664219; doi:10.3389/fendo.2024.1502050)
Supplement: Supplementary file 2 [file Table2.doc]

|  | Questions | Yes | No | Don't  Know Score |
| --- | --- | --- | --- | --- |
| 1 | Are there previous conclusive reports on this reactions? | +1 | 0 | 0 |
| 2 | Did the adverse event appear after the suspected drug? | +2 | -1 | 0 |
| 3 | Did the adverse reaction improve when the drug was discontinued, or a specific antagonist was administered? | +1 | 0 | 0 |
| 4 | Did the adverse reaction reappear when the drug was readministered? | +2 | -1 | 0 |
| 5 | Are there alternative causes (other than the drug) that could on their own have caused the reaction? | -1 | +2 | 0 |
| 6 | Did the reaction reappear when a placebo was given? | -1 | +1 | 0 |
| 7 | Was the drug detected in the blood (or other fluids) in concentrations known to be toxic? | +1 | 0 | 0 |
| 8 | Was the reaction more severe when the dose was increased or less severe when the dose was decreased? | +1 | 0 | 0 |
| 9 | Did the patient have a similar reaction to the same or similar drug in any previous exposure? | +1 | 0 | 0 |
| 10 | Was the adverse event confirmed by any objective evidence? | +1 | 0 | 0 |
| Total Score |  |  |  |  |
| >9=highly probable,5-8=probable, 1-4=possible and ≤0=doubtful | | | | |

Naranjo ADR Probability Scale
