# Supplementary material for: Risk factor screening and prediction modeling of gastrointestinal adverse reactions caused by GLP-1RAs
Source: Front Endocrinol (Lausanne). 2024 Dec 5;15:1502050. doi: 10.3389/fendo.2024.1502050 (PMC11664219; doi:10.3389/fendo.2024.1502050)
Supplement: Supplementary file 3 [file Table3.doc]

| Drug category | Antihyperglycemic drugs | Non-steroidal anti-inflammatory drugs | Antibacterial | Antipsychotic drugs | Lipid-modulating drugs | Antihypertensive drugs | Other drugs |
| --- | --- | --- | --- | --- | --- | --- | --- |
| Drug name | Metformin Hydrochloride, Pioglitazone metformin hydrochloride tablets, Hydrochloride, Acarbose, Voglibose, Gliquidone, Gliclazide, Glimepiride, Linagliptin, Pioglitazone hydrochloride, Sitagliptin phosphate, Vildagliptin, Saxagliptin, Dapagliflozin, Canagliflozin, Empagliflozin. | Aspirin, Ibuprofen, Aspirin enteric-coated tablets, Diclofenac sodium, Celecoxib | ClarithroMycin, Metronidazole, Amoxicillin, Ceminir capsules, Sulfamethoxazole, Moxifloxacin hydrochloride, Levofloxacin, Itraconazole, Cefaclor capsules. | Quetiapine Fumarate, Olanzapine, | Atorvastatin calcium, Rosuvastatin calcium, Fenofibrate, Probucol, simvastatin, Fluvastatin sodium capsules, Pitavastatin calcium. | Metoprolol succinate, Metoprolol tartrate, nifedipine, Valsartan amlodipine, Amlodipine besylate, Levoamlodipine maleate, Carvedilol, Metoprolol tartrate, Bisoprolol fumarate, Feilodipine, Benidipine hydrochloride, Nimodipine, lacidipine, Atenolol, Propranolol hydrochloride. | Sodium bicarbonate, Epalrestat, Clopidogrel Bisulfate, Sacubitril Valsartan Sodium, Citicoline Sodium Capsules, Lianhua Qingwen Capsule, Diammonium glycyrrhizinate, Lactulose Oral solution, Febuxostat, Suhuang Zhike, Suxiao jiuxin pills, Glutamine, Mosapride citrate, Sultamicillin tosylate, Compound paracetamol. |

Supplementary Table S3
